# Wnt signaling in triple-negative breast cancer

**DOI:** 10.1038/oncsis.2017.14

**Published:** 2017-04-03

**Authors:** SÖ-G Pohl, N Brook, M Agostino, F Arfuso, A P Kumar, A Dharmarajan

**Affiliations:** 1 Stem Cell and Cancer Biology Laboratory, Perth, WA, Australia; 2 School of Biomedical Sciences, Curtin University, Perth, WA, Australia; 3 Curtin Health Innovation Research Institute, Curtin University, Perth, WA, Australia; 4 Curtin Institute for Computation, Curtin University, Perth, WA, Australia; 5 Cancer Science Institute of Singapore, National University of Singapore, Singapore, Singapore; 6 Department of Pharmacology, Yong Loo Lin School of Medicine, National University of Singapore, Centre for Translational Medicine, Singapore, Singapore; 7 Department of Biological Sciences, University of North Texas, Denton, TX, USA

## Abstract

Wnt signaling regulates a variety of cellular processes, including cell fate, differentiation, proliferation and stem cell pluripotency. Aberrant Wnt signaling is a hallmark of many cancers. An aggressive subtype of breast cancer, known as triple-negative breast cancer (TNBC), demonstrates dysregulation in canonical and non-canonical Wnt signaling. In this review, we summarize regulators of canonical and non-canonical Wnt signaling, as well as Wnt signaling dysfunction that mediates the progression of TNBC. We review the complex molecular nature of TNBC and the emerging therapies that are currently under investigation for the treatment of this disease.

## Introduction

Breast cancer represents one of the most significant disease burdens of any cancer worldwide. Today, women have a one in eight chance of developing breast cancer over their lifetime, a risk that is significantly increased if they have inherited harmful mutations in *BRCA1* or *BRCA2.*^1^ However, breast cancer is a complex, heterogeneous disease characterized by a great multitude of aberrations at the genomic and molecular level, which can manifest in dysregulated signaling pathways. A hallmark of many cancers is aberrant regulation of the Wnt signaling pathway, and breast cancer is no exception.^2^

Triple-negative breast cancer (TNBC), an aggressive subtype of breast cancer with a poor prognosis,^3^ is characterized by tumors that do not express estrogen receptors (ERs) or progesterone receptors (PRs), nor display an overexpression of human epidermal growth factor receptor 2 (HER2).^4^ Therapies targeted against HER2-positive breast cancers, such as trastuzumab (Herceptin),^5^ and those targeted against ER-positive breast cancers, such as tamoxifen,^6^ have no therapeutic benefit to individuals with the TNBC subtype. Surgical intervention and chemotherapy have been the major treatment avenues for TNBC; however, recently developed small molecules and immunotherapeutics^7^ are showing promise. In this review, we will summarize the involvement of dysregulated Wnt signaling in the progression of TNBC and TNBC stem cells, as well as the emerging therapies that are currently under investigation.

## The Wnt signaling pathway

### The Wnt/β-catenin pathway (canonical pathway)

**Figure 1:**
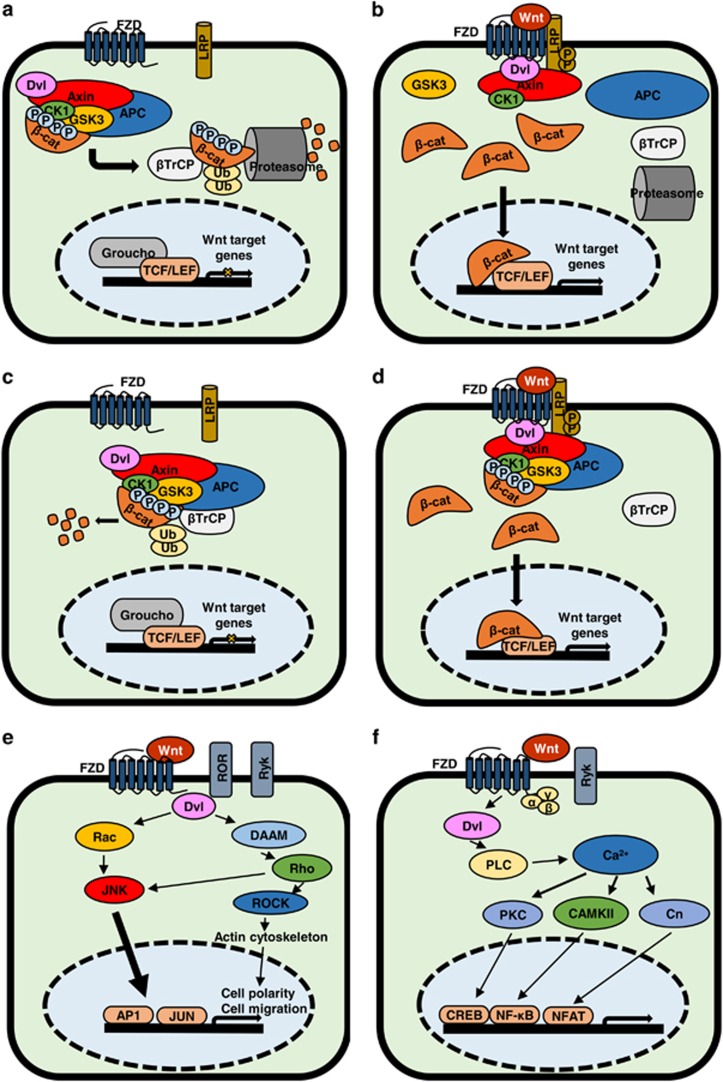
Classical and new Wnt/β-catenin pathway canonical and non-canonical pathways. (**a**) Overview of the ‘classical’ model of Wnt/β-catenin signaling in OFF state with no ligand bound to FZD receptor. (**b**) Overview of the ‘classical’ model of Wnt/β-catenin signaling pathway in ON state where Wnt ligand is bound to FZD receptor. (**c**) Overview of ‘new’ model of Wnt/β-catenin signaling in OFF state with no ligand bound to FZD receptor. (**d**) Overview of the ‘new’ model of Wnt/β-catenin signaling in ON state with Wnt ligand bound to FZD receptor. (**e**) Overview of Wnt planar cell polarity (PCP) pathway in ON state. Wnt binds multiple receptors including FZD and co-receptors ROR and Ryk. This activates Rho and Rac, which activate ROCK and c-Jun N-terminal kinase (JNK), respectively, leading to actin polymerization and regulates cytoskeletal arrangements. (**f**) Overview of Wnt/Ca^2+^ pathway in ON state. Wnt is able to bind FZD, Ryk to initiate signal transduction, which is effected through Dvl and G proteins (α, β, γ). Gene transcription is induced through proteins PKC, CaMKII and Cn (Calcineurin)-activating transcription factors.

There are currently two models of canonical Wnt/β-catenin signaling. In the classical model, the destruction complex remains intact in the absence of Wnt stimulation (Figure 1a). Casein kinase 1 (CK1) primes β-catenin for destruction by phosphorylating Ser45, which then allows activated Glycogen synthase kinase 3 (GSK3) to phosphorylate β-catenin at Ser33, Ser37 and Thr41.^8^ The phosphorylated residues of β-catenin interact with the β-propeller domain of the E3 ubiquitin ligase β-TrCP, which then ubiquitinates β-catenin, thus targeting it for proteosomal degradation.^9^ Wnt/β-catenin signaling is initiated by Wnt ligands binding to a Frizzled receptor (FZD), as well as the co-receptors low-density lipoproteins 5/6 (LRP5/6). This results in activation of FZD, permitting binding of Dishevelled (Dvl)^10^ and phosphorylation of one or more cytoplasmic motifs of LRP5/6. A single phosphorylated motif is sufficient to activate Wnt signaling.^11^ Phosphorylated LRP5/6 can then interact with Axin. This interaction destabilizes the β-catenin destruction complex, which requires Axin as a scaffold and contains Dvl, the serine–threonine kinases casein kinase 1α/β (CK1), glycogen synthase kinase 3α/β (GSK3) and adenomatous polyposis coli (APC).^12^ Destabilizing the destruction complex prevents phosphorylation of β-catenin, which then accumulates in the cytosol before translocating to the nucleus. Once there, it binds to Transcription factor/lymphoid enhancer-binding factor (TCF/LEF) transcription factors and displaces transcriptional repressor Groucho to initiate the transcription of Wnt target genes (Figure 1b).^13^ In the new model,^14, 15, 16^ the destruction complex is stabilized by Axin in both the presence and absence (Figure 1c) of Wnt activation, and β-catenin is degraded through phosphorylation-mediated recognition by β-TrCP in the intact complex. This allows newly synthesized β-catenin to accumulate in the cytosol before nuclear translocation (Figure 1d). This was demonstrated through co-immunoprecipitation, whereby β-catenin phosphorylated at Ser33/Ser37/Thr41 was shown to interact with the destruction complex upon Wnt activation, which also disrupted the interaction of β-TrCP with the Axin1-β–catenin complex.^15^ It has also been proposed that GSK3 inhibition, and thus β-catenin translocation after Wnt activation, is mediated through the sequestration of GSK3 inside multivesicular endosomes.^17^ This further demonstrates the complexity of Wnt signaling.

### Planar cell polarity pathway

The planar cell polarity pathway (Figure 1e) is a non-canonical, β-catenin-independent pathway that regulates cellular organization and polarity, partly through cytoskeletal organization.^18^ Wnt ligands, such as Wnt5a, bind to FZD receptors and co-receptors, including ROR,^19^ Ryk^20^ and PTK.^21^ Dvl interacts with Rac1^22^ and Dvl-associated activator of morphogenesis 1 (DAAM1). Rac1 activates c-Jun N-terminal kinase, leading to actin polymerization,^22^ while DAAM1 activates Rho, which in turn activates Rho-associated kinase (ROCK) to regulate cellular cytoskeletal arrangements.^23^

### The Wnt/Ca^2+^ pathway

The Wnt/Ca^2+^ pathway (Figure 1f) is activated through Wnt binding to FZD, which interacts with G proteins and Dvl.^24^ These interactions can activate cGMP-specific phosphodiesterase or phospholipase C, resulting in a release of intracellular calcium. This results in the activation of downstream signaling proteins PKC, calcineurin and CaMKII.^25^ CaMKII activates nuclear factor of activated T-cells, which can regulate cell adhesion and migration.^26^ Wnt5a induces activation of CaMKII-dependent Wnt/Ca^2+^ signaling. CaMKII phosphorylates transforming growth factor β-activated kinase, which activates Nemo-like kinase.^27^ This cascade antagonizes canonical Wnt/β-catenin signaling by Nemo-like kinase phosphorylation of TCF4 and prevents the β-catenin–TCF4 complex from binding to DNA.^28^

### Wnt ligands

**Figure 2:**
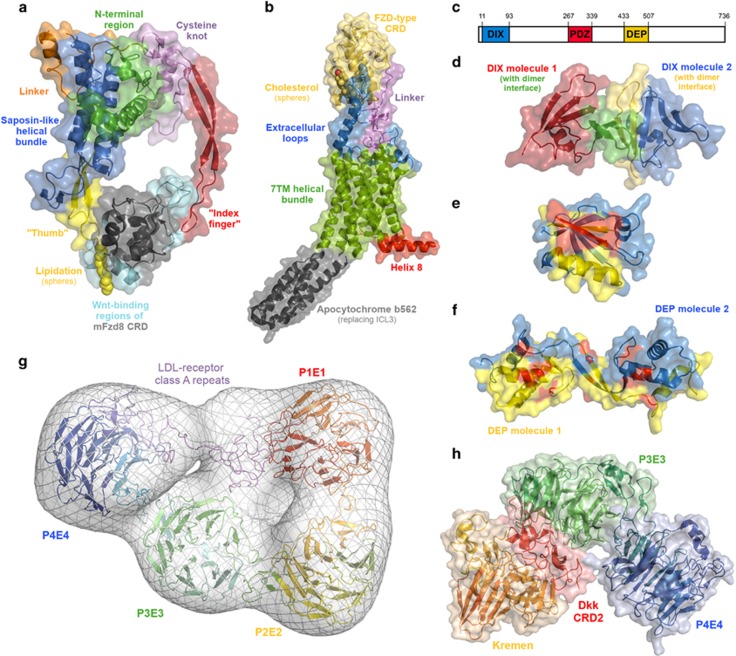
Molecular structures of the key Wnt signaling proteins and interactions. (**a**) X-ray crystal structure of the *Xenopus* Wnt8 complex with the mouse FZD8 cysteine-rich domain (PDB 4F0A). The key structural regions of the Wnt fold are highlighted, as are the major Wnt-interacting regions of the CRD. (**b**) X-ray crystal structure of the Smoothened receptor (PDB 5L7D), a Class F G protein-coupled receptor, related to FZD. The key structural regions of Smo are highlighted, as well as helix 8, which is of relevance for Dishevelled binding by FZD. (**c**) Schematic representation of the location of the DIX, PDZ and DEP domains within Dvl. (**d**) X-ray crystal structure of the DIX homodimer (PDB 4WIP). (**e**) X-ray crystal structure of the PDZ domain bound to a peptide (red; PDB 3CBX). The peptide-binding site is shown in yellow. (**f**) X-ray crystal structure of a DEP homodimer (PDB 5LNP), highlighting residues known to affect Wnt signaling (shown in red). (**g**) Model of the LRP6 ectodomain generated by molecular dynamics flexible fitting of the crystal structures of the P1E1–P2E2 domains (PDB 3S94) and P3E3–P4E4 domains (PDB 4A0P), and a homology model of the LDL-R type A domains (generated in Prime, based on the crystal structure of the LDL receptor ectodomain (PDB 1N7D)) to the electron microscopy structure (EMDatabank accession 1964). Gaps in the crystal structures and between the various components modeled using Prime. (**h**) X-ray crystal structure complex of the cysteine-rich domain 2 of Dickkopf with Kremen and the LRP6 P3E4–P4E4 domains (PDB 5FWW).

To date, 19 members of the Wnt family have been identified in mammals, all ranging between 350 and 400 amino acids in length and characterized by a conserved fold containing a conserved motif of 24 cysteine residues (Figure 2a).^29^ Wnt ligands are modified by lipidation, specifically, the addition of a palmitoleyl group to a conserved serine by the membrane-bound *O*-acyltransferase Porcupine.^30^ Wnt lipidation is crucial for secretion from the endoplasmic reticulum^31^ and essential for Wnt function. Wnt lipidation was initially suggested to occur at Cys77 of Wnt3a (cysteine 3 of the Wnt fold);^32^ however, lipidation at this cysteine has been conclusively disproven by crystallographic,^33^ mutational^31^ and imaging studies.^30^

### FZD receptors

FZD receptors are a group of 10 membrane proteins featuring an extracellular cysteine-rich domain (CRD) and a seven-transmembrane domain.^34^ Along with the Smoothened receptor (Smo), the FZDs comprise the family of Class F G protein-coupled receptors. The crystal structure of XWnt8 in complex with the mouse FZD8 CRD^33^ revealed an unusual interaction involving the direct binding of the Wnt lipid to a binding site on one side of the CRD (the ‘thumb’ region), as well as the binding of the region from cysteines 19 to 22 of XWnt8 to the other side of the CRD (the ‘index finger’ region; Figure 2a).^35^ Although no complete structures are available for any FZD, several structures of Smo are known,^36, 37, 38^ most recently including both the CRD and seven-transmembrane regions (Figure 2b),^39^ which are suggestive of the likely structure of FZD.

### Disheveled

Three Dvl homologs are known (Dvl1/2/3), sharing high overall sequence similarity.^40^ Dvl consists of three structurally defined domains: the DIX, PDZ and DEP domains. These three domains are separated by large insertions of unknown structure (Figure 2c); however, some functional significance has been ascribed to conserved sequences within the unstructured regions.^41^

Dvl polymerizes via the head-to-tail interaction of its DIX domain (Figure 2d). The DIX domain also mediates interaction with Axin.^42^ Mutations (V67A, K68A, Y27D) in the polymerization interface of the DIX domain strongly suppress Wnt signaling.^43^ The PDZ domain of Dvl (Figure 2e) interacts with a conserved motif in the FZD C-terminal (KTxxxW).^24^ The PDZ–FZD interaction is relatively weak, and is likely supplanted by interactions of the DEP domain with FZD. Greater insight in the role of the DEP domain in Wnt signaling was recently revealed, with this domain shown to bind as a monomer to FZD, then undergo subsequent domain swapping to assemble Wnt signalosomes. Furthermore, upon Wnt stimulation, DEP domain swapping initiates DIX-dependent Dvl and Axin polymerization, leading to the inhibition of GSK3 and Wnt signal transduction. Mutants (E499G, D460K, G436P, K438M, D449I and D452I) in the DEP domain strongly diminish Wnt signaling upon Wnt stimulation (Figure 2f).^44, 45^ Dvl has also been shown to promote ubiquitination-mediated FZD degradation by RNF43.^46^ This finding suggests a dual agonist/antagonist role for Dvl in Wnt signaling.

### Low-density lipoprotein receptor 5/6

The extracellular domain of LRPs consists of four β-propeller repeats interspersed with epidermal growth factor repeats, followed by three LRP type A repeats (Figure 2g).^10^ The majority of Wnts bind to the first β-propeller/epidermal growth factor repeat (P1E1–P2E2), although Wnt3 and Wnt 3a preferentially bind to the second repeat (P3E3–P3E4).^47^ Wnt3 and Wnt3a binding to LRPs is competitively inhibited by Dickkopf binding to LRP (Figure 2h).^48, 49, 50^ The intracellular action of LRP5/6 is less clearly understood, although it is known that Wnt activation initiates phosphorylation of the intracellular PPPSPxP motifs of LRP5/6 by GSK3 and CK1, allowing the recruitment of Axin.^51^ Importantly, it has also been shown that without the FZD–Dvl interaction, Wnt is unable to induce phosphorylation of LRP6, reinforcing the complex interplay of proteins involved in Wnt signaling.^24, 51^

### ROR family receptor tyrosine kinases

The ROR family of receptor tyrosine kinases consists of two evolutionarily conserved members, ROR1 and ROR2.^52^ The ROR ectodomains feature a FZD-type CRD most closely related to that of the skeletal muscle receptor tyrosine-protein kinase.^53^ ROR2 is involved in Wnt5a-mediated signaling; Wnt5a binding to ROR2 initiates ROR2 homodimerization, stimulating autophosphorylation at Tyr646.^54^ It has been demonstrated that Wnt5a and Wnt3a bind to ROR2; however, only Wnt5a is able to initiate the activation of the ROR2 signaling cascade.^55^ Recently, high expression of ROR1 has been demonstrated in TNBC cell lines, where it interacts with CK1ε to promote tumor survival and growth after stimulation with Wnt5a to activate phosphoinositide 3-kinase (PI3K)/AKT signaling.^56^

### DEAD-box helicases

**Figure 3:**
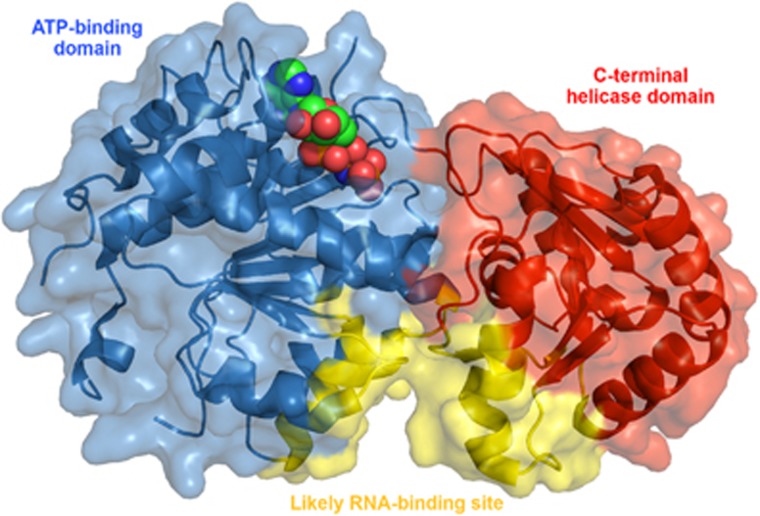
X-ray crystal structure of the ATP-binding and C-terminal helicase domains of the DEAD-box helicase DDX3 (PDB 5E7M). AMP-PNP, a non-hydrolyzable ATP analog, is shown in spheres in the ATP-binding site.

DEAD-box helicases (DDXs), named for a conserved amino-acid sequence in their ATP-binding domain (Asp-Glu-Ala-Asp), belong to a highly conserved family of ATP-dependent DNA/RNA helicases.^57^ They consist of a highly conserved helicase core with two domains, displaying high similarity to the recA bacterial DNA recombination protein (Figure 3).^58^ These multifunctional proteins have roles in translation initiation, pre- and post-translational modifications, DNA repair, microRNA (miR) processing, ribosome biogenesis and RNA decay.^59, 60, 61^ Furthermore, DDXs have been recently implicated in breast tumorigenesis and activation of cancer stem cell (CSC) stemness through various pathways, including Wnt.^62, 63^ DDXs can be regulated by β-catenin/TCF-driven transcription and have also been shown to regulate upstream Wnt signaling. The role of DDXs is discussed in further detail later in the section titled ‘DDXs, Wnt and TNBC’.

## Breast cancer subtypes

Breast cancer is a diverse and complex disease, broadly characterized by four molecularly distinct subtypes, including luminal A, luminal B, HER2-overexpressed and triple-negative breast cancer (TNBC).^64^ The luminal A subtype is characterized as ER/PR-positive and HER2-negative, expressing Bcl-2, cytokeratin 8/18 and low Ki67.^65^ Luminal B subtypes are more aggressive ER^+^ breast tumors, characterized as HER2^−^ with high Ki67, or HER2^+^, PR^−^ and ER^+^,^66^ with cyclin B1 overexpression.^67^ The HER2 subtype is characterized by amplification of the ERBB2/HER2 gene.^68^

TNBC, including basal-like and claudin-low subtypes, accounts for 10–20% of breast cancers and is characterized by a lack of PR, ER and HER2 overexpression.^69^ TNBC patients present with higher incidence of distant disease recurrence within 3 years of diagnosis, with a high frequency of visceral metastases.^70^ The prognosis for patients diagnosed with TNBC is poor, with patients who respond poorly to adjuvant treatment exhibiting worse outcomes.^4^

### TNBC subtypes

TNBC has been categorized into a number of distinct molecular subtypes; however, there remains much intertumoral mutational and transcriptional heterogeneity within these subtypes. The molecular heterogeneity of TNBC confounds the clinical approach to TNBC treatment. TNBCs are characterized by high clonal frequencies of single gene mutations in the key tumorigenesis driver genes, including *TP53*, *PIK3CA* and *PTEN*, indicating that clonal evolution of these mutated genes is an early event in TNBC development.^71^ However, mutation frequencies within these genes are not uniform among TNBC cases.^71, 72^

Lehmann *et al.*^73^ determined gene expression signatures in 587 TNBC cases from 21 breast cancer data sets and identified six molecularly distinct TNBC subtypes. These include basal-like 1, basal-like 2, immunomodulatory, mesenchymal (M), mesenchymal stem-like (MSL), and luminal androgen receptor (LAR). These subtypes, various gene ontology pathways and associated Wnt genes are described in Table 1. Recent RNA profiling performed by Burstein *et al.*^92^ showed overlap of LAR and MES subtypes based on Lehmann’s gene expression profiling, but was unable to reproduce all observations.^92^ The findings of both of these studies indicate the presence of at least four molecularly distinct and stable TNBC subtypes, defined as LAR, mesenchymal (MES), basal-like immune-suppressed (BLIS) and basal-like immune-activated (BLIA).^92^ Furthermore, these studies suggest molecular targets for the development of therapeutics specific to the treatment of TNBC.

### LAR subtype

The LAR subtype accounts for ∼10% of TNBCs, whereby tumor cells exhibit positive staining for androgen receptors (ARs) and are driven by AR signaling.^69, 73^ The LAR subtype of TNBC displays genomic amplification of *CCND1*, a gene regulated by the Wnt/β-catenin pathway.^92^ There is some discordance within the literature in regards to the prognostic utility of AR status, with studies indicating no significant effect on survival rates associated with AR expression,^93^ although AR^+^ TNBC individuals have been shown to have a positive clinical response to the nonsteroidal antiandrogen, bilcautamide.^94, 95^ In a study designed to test the benefit of tamoxifen on ER^−^ and TNBC patients, it was found that expression of AR^+^ versus AR^−^ individuals predicted a decreased recurrence rate and treatment benefit with AR^+^ patients;^96^ this is a result of tamoxifen exhibiting agonist activity on AR-expressing cells.^97^

### MES subtype

The MES subtype, encompassing Lehmann’s M, MSL and claudin-low subtypes, is characterized by the overexpression of genes associated with cellular motility, proliferation and growth signaling pathways.^73, 92^ MES subtypes have high expression of platelet-derived growth factor, insulin-like growth factor 1 and c-kit.^92^ MES tumors express mesenchymal stem cell markers, including the breast stem cell marker ALDH1A1, and are enriched in genes associated with epithelial–mesenchymal transition (EMT) and other stem-like properties.^73, 98^ Within Lehmann’s M and MSL subtypes, there are a number of enriched genes associated with EMT that are also modulated by Wnt signaling, including *MMP2, TWIST, SNAI2* and *TCF4.*^77^ A gene set involved in Wnt/β-catenin signaling in the M and MSL subtypes, including *CTNNB1* (β-catenin)*, DKK2, DKK3, SFRP4, TCF4, TCF7L2* and *FZD4*, was also found to be enriched.^73^ MES tumors are associated with a poorer distant metastasis-free survival at 5 years compared to other subtypes, likely associated with increased expression of cellular motility genes leading to increased metastasis.^73^

### BLIS subtype

BLIS is characterized as an immune-suppressed TNBC subtype with downregulated immune signaling pathways and reduced expression of immune function genes.^92, 99^ BLIS tumors exhibit enhanced expression of mitotic and cell cycle pathway genes, with overexpression of proliferative genes, including *CENPF*, *BUB1* and *PRC1,*^99^ Sry-related HMG box (SOX) transcription factors, and the immune-regulatory molecule V-domain-containing T-cell activation inhibitor.^92^ SOX transcription factors share a closely related consensus binding sequence to TCF/LEF transcription factors^100^ and are known modulators of Wnt/β-catenin signaling.^101^ Survival analysis shows that patients with the BLIS subtype TNBC experience lower rates of recurrence-free survival compared to other TNBC subtypes.^99^

### BLIA subtype

The BLIA subtype is characterized by upregulation of immune activating pathways, with overexpression of STAT transcription factors and cytotoxic T-lymphocyte-associated protein 4.^92^ Furthermore, the BLIA subtype demonstrates amplification of *CDK1*, which was recently found to phosphorylate the Wnt regulator TAZ.^92, 102^ BLIA tumors have increased levels of lymphocytic infiltration and are thus associated with improved disease-free survival rates and patient outcomes compared to other TNBC subtypes, although still associated with a relatively high risk of recurrence (~20%).^92, 103^

### Wnt dysregulation in TNBC and TNBC stem cells

Aberrant Wnt signaling is a characteristic of TNBC, with both canonical and non-canonical pathways implicated in TNBC tumorigenesis^104, 105^ and metastasis.^106^ Enrichment of Wnt/β-catenin signaling is evident in TNBC and is associated with poor clinical outcomes within this subtype.^107, 108^ TNBC patients displaying dysregulated Wnt/β-catenin signaling are more likely to develop lung and brain secondary metastases.^106^ Studies have shown that nuclear accumulation of β-catenin promotes cell migration, colony formation, stem-like features and chemoresistance of TNBC cells *in vitro* and TNBC tumorigenesis in mouse cancer models, thus suggesting that canonical Wnt signaling is a major driving force of TNBC tumorigenesis.^104^ Although the Wnt/β-catenin pathway is associated with the clinicopathological features of TNBC, this is not due to *CTNBB1* mutations.^108^ Studies have also implicated dysregulation of non-canonical Wnt signaling pathways in the highly metastatic behavior of TNBC cells and CSCs, specifically through aberrant c-Jun N-terminal kinase activation.^109^

CSCs, or cancer stem-like cells, are a small subset of cells within the heterogeneous tumor bulk that are thought to be responsible for tumor initiation.^110^ These cells also have intrinsic mechanisms for chemoresistance, such as upregulation of drug transporters, including the breast cancer resistance protein (also known as ABCG^2^).^111^ By evading the standard chemotherapeutic treatments, it is thought that the CSCs are also responsible for the relapse experienced in many cancers, especially TNBCs.^112^ Studies have also shown that these cells are a main contributor to metastasis, and are able to initiate solid tumor formation when xenotransplanted at low cell densities.^113^ TNBC stem cells are isolated from tumors as CD44^+^ (homing cell adhesion molecule), CD24^−^ (heat stable antigen), CD49f^+^ cells.^114^ CSCs also differ metabolically to other cancer cells. They are more reliant on mitochondrial respiration, which is supported by their higher mitochondrial reactive oxygen species, enhanced oxygen consumption and higher mitochondrial mass, allowing for features such as resistance to DNA damage.^115^

Wnt signaling is essential for normal breast stem cell function and mammary gland development during embryogenesis, postnatal development and pregnancy,^116^ with adult mammary glands containing Wnt-responsive stem cell populations.^117^ Studies have shown that aberrant Wnt signaling in breast cancer stem cells (BCSCs) is a key event in breast tumorigenesis.^118^ Wnt/β-catenin signaling has been linked to TNBC tumorigenesis by regulating the key tumor-associated characteristics, including migration, stemness, proliferation and chemoresistance in TNBC cells and CSCs.^104^ A recent study has also demonstrated that Wnt/β-catenin signaling activity is higher in breast CSCs than the bulk tumor population, based on β-catenin, TCF4 and LEF1 expression in Aldefluor-positive cells versus Aldefluor-negative cells.^119^ Treatment with Wnt3a increased the number of ALDH^+^ breast CSCs, and knockdown of Wnt1 reduced the tumor-forming efficiency of breast CSCs *in vitro*.^119^

**Figure 4:**
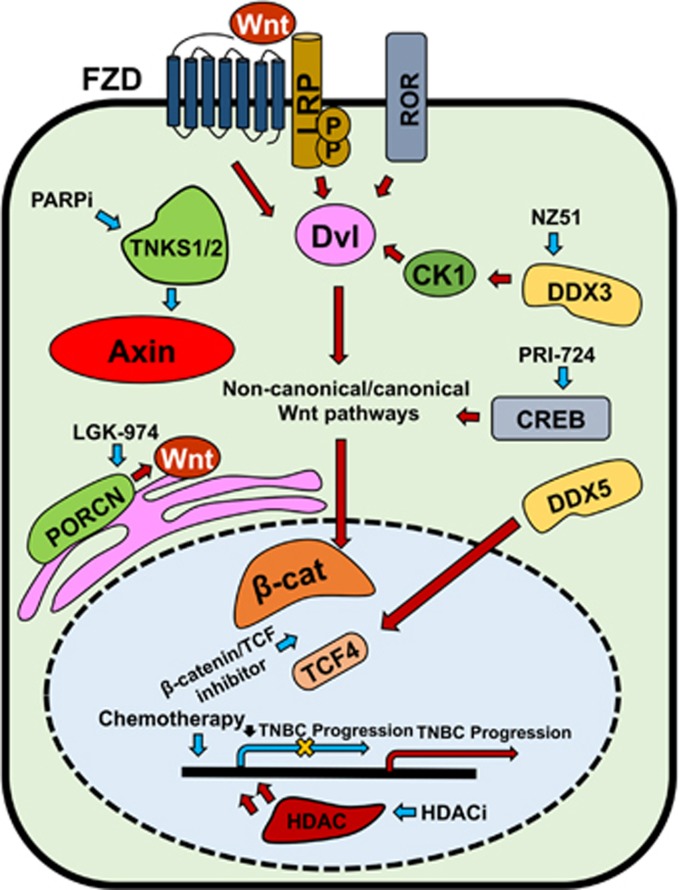
Overview of Wnt signaling regulators contributing to TNBC progression and their targeted therapies. Canonical and non-canonical Wnt pathways are activated through Fzd, LRP and ROR receptors. Blue arrows indicate suppression/inhibition of Wnt regulators and pathways (with a net result of downregulation of Wnt target gene transcription, indicated by yellow cross); red arrows indicate activation of Wnt regulators and pathways.

Furthermore, studies have shown that Wnt-derived breast tumors are maintained by clones capable of re-activating Wnt overexpression post-Wnt inhibition, indicating that aberrant Wnt activation is a key driver of breast cancer recurrence and progression.^120^ A recent review highlighted the potential importance of Wnt/β-catenin signaling, along with other developmental signaling pathways, including Cripto-1 and Notch/CSL, in the regulation of TNBC stem cells and therapy resistance in TNBC.^121^ An overview of Wnt signaling dysregulation is given in Figure 4.

### FZDs in TNBC

#### FZD6

FZD6 exhibits increased gene copy number variations and overexpression in breast cancers. This is more frequent in TNBC than ER^+^ tumors. A study by Corda *et al.*^122^ determined that FZD6 was involved in the regulation of cell motility, invasion and three-dimensional (3D) growth, although it did not regulate proliferation in TNBC. This was confirmed by a significant reduction in distant metastases detected in various organs *in vivo* after the injection of MDA-MB-231 cells depleted of FZD6. Short hairpin RNA directed at *FZD6* *in vitro* was found to reduce cell invasion through a reduction in active Rho and the subsequent reduction in fibronectin fibres. This indicated that FZD6 regulates cell motility and invasion through non-canonical Wnt signaling. This study also suggests that FZD6 overexpression in TNBC has a high prognostic value in determining the risk of metastasis.^122^

#### FZD7

Microarray analysis determined that FZD7 expression is upregulated in TNBC tissue and cell lines, and promotes tumorigenesis via canonical Wnt signaling pathways.^123^ Short hairpin RNA-mediated silencing of FZD7 reduced invasiveness and colony formation in TNBC cell lines.^123^ A recent study found that ΔNp63, an isoform of Transformation-related protein 63 (p63), enhanced FZD7 expression and increased Wnt signaling in TNBC tumor tissue and cell lines.^124^

Aberrant FZD7 expression is implicated in TNBC stem cell-mediated tumorigenesis. A study recently found that knockdown of ΔNp63 in TNBC cell lines decreased FZD7 expression and tumorsphere formation, indicating that ΔNp63/FZD7 upregulation induced TNBC stem cells and promoted tumor formation in TNBC.^124^ The findings of this study highlight the potential clinical importance of ΔNp63/FZD7-Wnt signaling in TNBC stem cells as a key driver of tumorigenesis and progression of TNBC.^124, 125^

#### FZD8

Gene expression studies have recently linked FZD8-driven Wnt signaling to chemoresistance in TNBC cell lines and TNBC stem cells. Treatment with cisplatin and tumor necrosis factor-related apoptosis-inducing ligand (TRAIL) in TNBC cell lines resulted in increased FZD8 expression in residual tumors of xenograft models.^126^ Furthermore, FZD8 silencing led to increased Wnt pathway-driven TNBC cell apoptosis *in vitro* and *in vivo.*^126^ The study showed that treatment with TRAIL/cisplatin increased expression of LEF-1 and TCF-7 in residual TNBC stem cells, thus implicating upregulation of Wnt signaling components in the development of chemoresistance.^126^ An inverse correlation between FZD8 and miR-100 was shown, where decreased miR-100 expression was linked to increased FZD8 expression and Wnt signaling, resulting in increased loco-regional breast cancer metastasis.^126, 127^ The role of miRs in Wnt signaling and TNBC is discussed in further detail below. c-Myc overexpression has been linked to FZD8 overexpression in TNBC cell lines, associating c-Myc-driven transcription to chemoresistance and TNBC CSC survival.^128^

### LRP5/6 in TNBC

LRP5/6 are essential for normal mammary development by regulating breast stem cell activity and are linked to basal-derived breast tumorigenesis.^129, 130, 131^ Studies in transgenic mice indicated that LRP5 knockdown led to resistance to Wnt1-induced tumor formation.^130^

Gene expression analyses found that LRP6 is overexpressed in human TNBC.^123, 131^ *In vivo* studies have shown that LRP6 silencing inhibited tumor growth in TNBC cell line-derived xenograft models.^132^ LRP6 and Wnt target gene *SOX9* have been shown to influence regulation of one another in TNBC cell lines. LRP6 overexpression led to *SOX9* upregulation, while knockdown of *SOX9* reduced LRP6 transcription and decreased cell invasion and proliferation.^133^

LRP6 overexpression led to the upregulation of Wnt signaling and was associated with increased stemness in TNBC cells.^134^ CD138 (Syndecan-1) is an EMT marker associated with both development and breast tumorigenesis,^135^ and has been shown to modulate TNBC stem cell properties by targeting Wnt signaling.^134^ Ibrahim *et al.*^134^ showed that CD138 modulates Wnt signaling in TNBC stem cells through LRP6, whereby CD138 silencing resulted in downregulated LRP6 expression and Wnt signaling.^134^

### RORs in TNBC

Primary breast cancer DNA microarray data set analysis has shown that ROR1 is expressed on breast cancer cells and absent in normal breast cells, with high ROR1 expression associated with poorer survival.^136^ Furthermore, the study showed that ROR1 silencing in TNBC cell lines increased apoptosis and reduced cell growth. High ROR1 expression in breast cancer cells is associated with high expression of EMT gene profiles and high incidences of disease recurrence and progression.^137^ ROR1 knockdown in TNBC cell lines resulted in reduced EMT-associated protein expression, reduced cell migration and invasion *in vitro*, and inhibited metastasis in xenograft models.^137^ ROR2 expression is present in both TNBC and non-TNBC, with ROR2^+^ TNBC patients exhibiting poorer survival outcomes compared to other subtypes.^138^ ROR2 knockdown in TNBC cell lines inhibited Wnt signaling and reduced TCF/LEF transcription.^138^ These findings indicate the potential prognostic and therapeutic significance of high ROR1/2 expression in TNBC.

### DDXs, WNT and TNBC

#### DDX3

DDX3 is a regulator of Wnt/β-catenin signaling, where it interacts with and increases the kinase activity of casein kinase 1ε and is required for the phosphorylation of Dvl2.^139^ It is known to have an oncogenic role in breast cancer, where non-tumorigenic MCF10A cell lines overexpressing DDX3 showed increased EMT, motility and invasiveness.^140^ The same study demonstrated that DDX3 expression was positively correlated with a more aggressive phenotype, and was highly expressed in TNBC cell lines. DDX3 overexpression resulted in E-cadherin downregulation and subsequent nuclear β-catenin translocation.^140^ Similarly, DDX3 inhibition by NZ51, a ring-expanded nucleoside analog that is predicted to bind to the ATP-binding site of DDX3, led to decreased proliferation, motility and invasiveness in TNBC cell lines and reduced tumor load and metastatic burden in preclinical *in vivo* models.^141^

#### DDX5 (p68)

DDX5 acts as a co-activator of Wnt/β-catenin signaling through regulation of TCF4 expression. In turn, β-catenin/TCF4 regulates DDX5 expression, forming a positive feedback loop associated with increased EMT marker expression in TNBC cells.^142^ DDX5 is thought to regulate p53-mediated repair of DNA damage, and DDX5 overexpression contributes to tumorigenesis and progression in breast cancers.^143^ DDX5 is highly expressed in basal-like breast cancers compared to luminal-like, and correlates with high EGFR and Ki67 expression in TNBC tissue.^144^ Furthermore, the study found that DDX5 regulates the expression of miR-21 and miR-182 in basal breast cancers, and is associated with malignant disease.

## The roles of miRs in Wnt signaling and TNBC

miRs are endogenous, short, non-coding RNA molecules that regulate cancer-related genes at the post-transcriptional level.^145^ miRs are differentially expressed in BCSCs and cancer cells, indicating that breast cancer-specific miRs are important in maintaining stemness and promoting tumorigenesis in BCSCs.^146^ Twenty-seven miRs differentially expressed in locally advanced TNBC have been previously identified, with many of these predicted to be involved in regulation of Wnt signaling pathway genes.^147, 148^

miR-374a overexpression led to suppression of Wnt pathway inhibiting components (PTEN and WIF1) and ultimately increased Wnt-mediated EMT and metastasis in multiple TNBC cell lines.^149^

miR-340 is downregulated in TNBC cell lines and has been linked with TNBC tumorigenesis regulation in multiple studies.^150^ Induction of miR-340 resulted in downregulation of Wnt pathway target genes (*CTNNB1*, *MYC* and *ROCK1*), decreased proliferation and increased apoptosis in a metastatic TNBC cell line. The study showed that miR-340 overexpression reduced cell motility and invasiveness, indicating that miR-340 has a fundamental role in regulating breast metastases.^151^ Another study recently found that induction of miR-340 in TNBC cell lines led to reduced expression of *SOX2*, an oncogene associated with the canonical Wnt signaling pathway.^152^

A study by Isobe *et al.*^153^ found that miR-142 upregulation is associated with BCSCs and activates canonical Wnt signaling by promoting APC breakdown in TNBC cell lines. The study found that miR-142 expression activated canonical Wnt signaling, leading to increased miR-150 expression, thereby contributing to breast tissue hyperproliferation, BCSC proliferation and reducing apoptosis in TNBC cell lines.^153^

A recent study has shown that miR-218-5p expression was significantly increased in TNBC, as well as bone metastases, from breast cancer patients.^154^ Anti-miR-218-5p led to a reduction in cell proliferation *in vitro* and decreased tumor growth, active osteoclasts and osteolytic lesions *in vivo*, while the opposite was seen with transfection of miR-218-5p. The miR was also shown to directly modulate Wnt/β-catenin signaling by binding to secreted FZD-related protein 1 and SOST. Anti-miR-218-5p suppressed Wnt signaling, which downregulated Parathyroid hormone-related protein expression, reducing breast cancer-induced osteolytic disease.^154^

## Current and emerging therapies for TNBC and TNBC stem cells

Systemic cytotoxic chemotherapy is clinically indicated in early TNBC and is associated with a greater treatment benefit than hormone receptor-positive tumors.^155^ Numerous early-phase clinical trials are currently underway, investigating various targeted molecules and combination therapies for the treatment of TNBC. In this section, we review current and emerging small molecule therapeutics for the treatment of TNBC (Figure 4); immunotherapeutics are reviewed elsewhere.^7^

### Chemotherapy

Anthracycline/taxane-based regimens are currently the standard of care in the treatment of adjuvant and neoadjuvant TNBC. However, a recent *in vitro* study has shown that treatment with docetaxel or doxorubicin had transient and negligible impact on cell growth in two TNBC cell lines, respectively. Furthermore, the study found that docetaxel and doxorubicin treatment resulted in deregulation of genes associated with stemness in TNBC cells.^156^ Molecular analysis found that doxorubicin treatment deregulated stem cell signaling pathways associated with cell growth, renewal and differentiation, with altered gene expression demonstrated in components of the Wnt signaling pathway, including FZD2, FZD4, FZD5, FZD6, FZD7, FZD9, Axin1, Wnt11, Wnt10a and Wnt5a. As such, the study concluded that docetaxel and doxorubicin induce stemness in differentiated TNBC cells, which likely accounts for acquired chemoresistance seen in refractory TNBC tumors.^156^

### Platinum agents

Platinum-based chemotherapeutics are a class of DNA-damaging agents, including cisplatin, carboplatin and oxaliplatin; these have established efficacy in breast cancer treatment.^157^ *In vitro* studies have indicated that combining TRAIL and cisplatin significantly increased BCSC death compared to other standard of care treatments in TNBC cell lines.^158^ The study showed that treatment with TRAIL and cisplatin inhibited Wnt1-mediated signaling and expression of cyclin D1, as well as the phosphorylation of β-catenin. Combination treatment with cisplatin and TRAIL also enhanced apoptosis, and inhibited proliferation and tumorsphere formation.^158^

### Wnt signaling inhibitors

Treatment with the small molecule β-catenin/TCF inhibitor CWP232228 inhibited β-catenin-mediated transcription, leading to inhibition of stem cell proliferation and reduction in tumor bulk in TNBC cell lines and TNBC patient-derived xenograft models, respectively.^159^ PRI-724, a CREB-binding protein inhibitor, and LGK-974, a Porcupine inhibitor, are two small molecules currently undergoing clinical development. Both molecules are currently under investigation for single agent use in ongoing phase I clinical trials in TNBC patients,^160^ with interim results yet to be released. Recent *in vitro* studies have shown that LGK-974 in combination with the PI3K/AKT/mTOR inhibitor BKM120 worked synergistically to decrease cell viability and enhance antitumor efficacy in TNBC cell lines.^161^

### PARP inhibitors

Poly (ADP-ribose) polymerase (PARP) is an enzyme involved in DNA repair mechanisms necessary for maintaining BRCA-mutated cell viability.^162, 163^ Included in the PARP enzyme family are tankyrase (TNKS)-1 and TNKS2. TNKS1 and TNKS2 are regulators of Wnt signaling through their interaction with Axin.^164, 165^ TNBCs share phenotypic characteristics with BRCA-mutated cancers, thus providing support for the use of PARP inhibitors.^166^ The small molecule TNKS1/2 inhibitor XAV939 showed effectiveness in the destabilization of Axin and reduction of Wnt activity, although data suggest that a combination approach may be more beneficial.^165^ Clinical trials evaluating the oral PARP inhibitor olaparib in BRCA1/2-positive metastatic breast cancer are currently underway, with interim results showing efficacy.^167^ Veliparib is another PARP inhibitor currently being evaluated in combination with paclitaxel and carboplatin for metastatic TNBC.^168^ Data from Phase I clinical trials of veliparib show acceptable safety, tolerance and good antineoplastic activity.^168^

### Histone deacetylase inhibitors

Histone deacetylase (HDAC) inhibitors are emerging as promising anti-TNBC agents because of their multifunctional capacity to regulate gene expression, cell growth and survival, as well as their ability to restore cellular aberrations due to epigenetic effects.^169^ Entinostat is an HDAC inhibitor recently shown to have anti-CSC effects in TNBC stem cells. An *in vivo* study found that entinostat treatment reduced TNBC stem cell populations, tumorsphere formation and miR-181a expression in TNBC cell lines.^170^ Furthermore, the study found that entinostat treatment in TNBC patient-derived xenografts reduced tumor growth and inhibited the development of lung metastases.^170^ Further *in vivo* studies have shown that triple therapy, combining entinostat, all-*trans* retinoic acid and doxorubicin, induced apoptosis of TNBC stem cells in culture and induced differentiation of TNBC CSCs both *in vitro* and *in vivo.*^171^ Panobinostat (LBH589) decreased cell survival and cell cycle progression at the G2/M stage in TNBC cell lines and *in vivo*. It also increased acetylation of the histones H3 (Lys3) and H4 (Lys8).^169^ Treatment with panobinostat upregulated cadherin-1 (CDH1) and reversed the M phenotype; CDH1 has been identified as a Wnt-signaling component in invasive breast carcinoma.^172^ An *in vivo* study found that salinomycin, a compound that selectively inhibits CSCs,^173^ in combination with panobinostat, significantly inhibited the growth of TNBC stem cells in TNBC patient-derived xenografts. The study found that salinomycin and panobinostat worked synergistically to inhibit cell cycle progression, enhance apoptosis and regulate EMT in TNBC stem cells.^173^

## Conclusions

The dysregulation of Wnt signaling is synonymous with cancer. TNBC is an aggressive, highly proliferative phenotype, which is characteristic of overactive signaling pathways. The accelerated development of sequencing technologies has allowed us to characterize the highly heterogeneous molecular landscape of TNBC with unprecedented detail. These technologies have allowed the discovery of new potential therapeutic targets, as well as to suggest where existing drugs may be of therapeutic value, for instance, in the use of tamoxifen on AR-positive TNBC patients. Like TNBC, Wnt signaling is highly complex and not yet fully characterized. The discovery of novel regulators in TNBC, such as DDXs, adds to the complexity, but also presents exciting new opportunities for the development of potential therapeutic targets. Structural knowledge of Wnt pathway proteins and interactions has expanded in recent years, providing opportunities for rational/structure-based drug design of novel cancer therapeutics.

## Figures and Tables

**Table 1:** Summary of the six molecular subtypes of TNBC characterized by Lehmann *et al.* ^73^

*TNBC subtype*	*Gene ontology pathway (GOP)*	*Genes found in GOPs with Wnt association*
Luminal androgen receptor (LAR)	Steroid pathway	*FKBP5*^74^
	Androgen metabolism	*SPDEF*^75^
	Fatty-acid synthesis	*FASN*^76^
Mesenchymal (M)/mesenchymal stem-like (MSL)	EMT	*MMP2,*^77^ *SNAI2,*^77^ *TCF4,*^77^ *TWIST1,*^77^ *ZEB1*^78^
	Wnt/β-catenin signaling	*CTNNB1,*^73^ *DKK2/3,*^73^ *TCF4,*^73^ *TCF7L2,*^73^ *CCND2,*^73^ *FZD4,*^73^ *CAV1,*^73^ *CAV2*^73^
Basal 1 (BL1)/Basal 2 (BL2)	DNA damage	*CHEK1,*^79^ *FANCA,*^80^ *FANCG,*^80^ *MSH2,*^81^ *RAD21*^82^
	Proliferation/cell cycle	*AURKB,*^83^ *PLK1,*^84^ *CENPA,*^84^ *BUB1,*^84^ *CCNA2,*^85^ *PRC1,*^86^ *MYC,*^87^ *NRAS*^88^
Immunomodulatory (IM)	JAK/STAT cytokine pathway	*CCR2,*^89^ *CCR5*^90^
	IL7 pathway	*IL7*^91^

Abbreviations: EMT, epithelial–mesenchymal transition; IL, interleukin; TNBC, triple-negative breast cancer.

Various gene ontology pathways were found to be enriched in the LAR, MSL, BL1, BL2 and IM subtypes. Analysis of the genes enriched in these pathways identified genes associated with Wnt signaling.
